# Visfatin Enhances RANKL-Induced Osteoclastogenesis In Vitro: Synergistic Interactions and Its Role as a Mediator in Osteoclast Differentiation and Activation

**DOI:** 10.3390/biom14121500

**Published:** 2024-11-25

**Authors:** Chang Youp Ok, Ryuk Jun Kwon, Hye-Ock Jang, Moon-Kyoung Bae, Soo-Kyung Bae

**Affiliations:** 1 Department of Dental Pharmacology, School of Dentistry, Education and Research Team for Life Science on Dentistry, Pusan National University, Yangsan 50612, Republic of Korea; luriel@hanmail.net (C.Y.O.); jho9612@pusan.ac.kr (H.-O.J.); 2 Periodontal Disease Signaling Network Research Center, School of Dentistry, Pusan National University, Yangsan 50612, Republic of Korea; 3 Family Medicine Clinic and Research Institute of Convergence of Biomedical Science and Technology, Pusan National University Yangsan Hospital, Yangsan 50612, Republic of Korea; brain6@hanmail.net; 4 Department of Oral Physiology, School of Dentistry, Education and Research Team for Life Science on Dentistry, Pusan National University, Yangsan 50612, Republic of Korea; mkbae@pusan.ac.kr; 5 Dental and Life Science Institute, School of Dentistry, Pusan National University, Yangsan 50612, Republic of Korea

**Keywords:** visfatin, RANKL, osteoclastogenesis, bone marrow-derived macrophages

## Abstract

Visfatin, an adipokine secreted by various cell types, plays multifaceted pathophysiological roles in inflammatory conditions, including obesity, which is closely associated with osteoclastogenesis, a key process underlying bone loss and increased osteoporosis (OP) risk. However, the role of visfatin in osteoclastogenesis remains controversial. This study was conducted to investigate the effects of visfatin on receptor activator of nuclear factor kappa-B ligand (RANKL)-induced osteoclast differentiation from precursor cells in vitro. Our results demonstrated that although visfatin exhibited a modest osteoclast-inductive effect relative to that of RANKL, co-stimulation of bone marrow-derived macrophages (BMDMs) with visfatin and RANKL led to significantly enhanced osteoclast differentiation and activation compared to individual stimulation. Neutralization of visfatin activity using blocking antibodies before differentiation markedly suppressed RANKL-induced osteoclastogenesis, as evidenced by a near-complete absence of tartrate-resistant acid phosphatase-positive multinucleated osteoclasts, decreased levels of nuclear factor of activated T cells cytoplasmic 1 and osteoclast-specific proteins, inhibition of nuclear factor-κB and mitogen-activated protein kinase signaling pathways, and a decrease in resorption pit formation. Our findings underscore the critical role of visfatin in RANKL-induced osteoclastogenesis in vitro and highlight the RANKL/visfatin signaling axis as a potential therapeutic target for destructive bone loss-related diseases.

## 1. Introduction

Bone homeostasis refers to the dynamic process by which bone tissue maintains an equilibrium through a coordinated balance between bone formation and resorption [1,2]. Bone tissue is predominantly synthesized by osteoblasts, which are specialized cells responsible for the formation and mineralization of new bone tissue. Conversely, bone resorption is mediated by osteoclasts, which are multinucleated cells that break down bone minerals and other inorganic components, subsequently releasing them into the bloodstream. Under normal physiological conditions, the activities of osteoclasts and osteoblasts are closely interconnected, facilitating the complete replacement of eroded bone with newly formed bone tissue [2]. However, a disruption in this homeostatic balance, favoring increased osteoclast formation and activity, may result in bone loss-related pathologies such as rheumatoid arthritis (RA), osteoarthritis, periodontitis, and osteoporosis (OP) [3,4,5].

Osteoclast differentiation refers to the process by which mononuclear hematopoietic precursors undergo fusion to form large, multinucleated cells [6]. This differentiation is initiated by the receptor activator of nuclear factor kappa-B ligand (RANKL), which is primarily expressed by osteoblasts and specific cells within bone tissue, as well as by lymphocytes, activated T cells, and some activated macrophages [7,8,9,10]. RANKL binds to its receptor, RANK, located on osteoclast precursor cells, thereby triggering a series of signaling events crucial for osteoclastogenesis. Following RANKL binding, intracellular signaling pathways, such as nuclear factor-κB (NF-κB) and mitogen-activated protein kinase (MAPK) pathways, are activated, which subsequently trigger downstream transcription factors such as nuclear factor of activated T cells cytoplasmic 1 (NFATc1), thereby promoting osteoclast differentiation. RANKL-induced osteoclastogenesis is recognized as a key canonical pathway, often described as a RANKL-dependent mechanism [9]. Notably, this process can be inhibited by osteoprotegerin (OPG), which acts as a soluble decoy receptor for RANKL, competing with RANK for binding, thereby blocking RANKL-induced osteoclastogenesis [7,9,11]. Additionally, various other molecules, including inflammatory cytokines, have been demonstrated to functionally substitute for RANKL in promoting osteoclastogenesis from hematopoietic precursors, representing non-canonical pathways of osteoclastogenesis [12,13]. These factors have been associated with pathological bone loss in diseases such as OP [1,12,13]. Therefore, identifying the additional factors involved in osteoclastogenesis and elucidating their underlying molecular mechanisms is imperative.

Osteoporosis and obesity are two prevalent conditions that significantly affect public health; however, their relationship is both complex and multifaceted [14,15]. Although obesity is traditionally considered to confer a mechanical protective effect on bone density owing to increased body weight, it is also associated with chronic inflammation and altered production of inflammatory adipokines—bioactive signaling proteins secreted by adipose tissue—that can affect bone metabolism [15,16]. For instance, an increase in the number of marrow adipocytes, commonly observed in conditions such as obesity, leads to the upregulation of RANKL expression, suggesting that RANKL may function as an adipokine [10]. In addition, elevated leptin and resistin levels in individuals with obesity have been demonstrated to disrupt the balance between bone formation and resorption, potentially increasing the risk of OP [17,18]. Conversely, the levels of adiponectin, which is typically regarded as protective against bone loss, are often diminished in obesity, potentially facilitating increased osteoclastogenesis, as reduced levels of adiponectin correlate with enhanced bone resorption [19,20,21]. Therefore, understanding the intricate interplay among these factors is essential for developing effective strategies to prevent and manage OP within the context of obesity.

Visfatin, also known as nicotinamide phosphoribosyltransferase (NAMPT) or pre-B-cell colony-enhancing factor, is a multifunctional protein that plays significant roles in cellular metabolism, immunity, and stress response [22,23]. As an intracellular enzyme, NAMPT catalyzes the conversion of nicotinamide to nicotinamide mononucleotide (NMN), a precursor of NAD+, which is essential for DNA repair, mitochondrial function, and energy metabolism [22,23]. Interestingly, intracellular NAMPT (iNAMPT) is secreted into the extracellular space as extracellular visfatin, where it acts as a growth factor, cytokine, or adipokine [22,23,24]. Despite its wide-ranging effects, the mechanisms behind visfatin secretion and its specific receptor are still unclear. It is suggested that visfatin exerts its actions by binding to cell surface receptors, potentially including the insulin receptor (IR) or Toll-Like Receptor 4 (TLR4) [25,26,27]. Initially recognized as a regulator of glucose metabolism and insulin sensitivity, visfatin has garnered significant interest owing to its diverse pathophysiological roles that extend beyond metabolic functions [22,23,24]. Increasing evidence, including our findings, indicates that visfatin functions as an inflammatory cytokine in various inflammatory conditions, such as cancer, type 2 diabetes, cardiovascular diseases, senescence/aging, periodontitis, and obesity [22,23,24,28,29,30,31,32,33,34]. Notably, visfatin levels are elevated in diseases associated with bone resorption, such as rheumatoid arthritis, osteoarthritis, periodontitis, and OP [33,35,36]. Given this, visfatin is expected to contribute to osteoclast differentiation and subsequent bone resorption. However, contrary to these expectations, visfatin (eNAMPT) has been reported to inhibit osteoclast differentiation [37]. On the other hand, recent studies on iNAMPT suggest that it may promote osteoclast differentiation [36]. Specifically, attenuated osteoclastogenesis was observed in iNAMPT-deficient primary BMMs and RAW 264.7 cells, whereas overexpression of iNAMPT enhanced osteoclast formation [36]. Therefore, the precise role of visfatin (eNAMPT) in osteoclastogenesis remains unclear and warrants further investigation.

In this study, we aimed to investigate the effects of visfatin on osteoclast differentiation and activation, particularly on RANKL-induced osteoclastogenesis, in bone marrow-derived macrophages (BMDMs) and explore the underlying mechanisms involved. Elucidating the intricate interplay between RANKL signaling and its modulation by factors such as visfatin is critical for understanding its contributions to bone metabolism and for developing targeted therapies for bone loss-related disorders.

## 2. Materials and Methods

### 2.1. Antibodies and Reagents

The following antibodies were used in this study: β-Actin (Abcam, Cambridge, MA, USA); dendritic cell-specific transmembrane protein (DC-STAMP) (Novus, Centennial, CO, USA); Cathepsin K (Biovision, Milpitas, CA, USA); visfatin, NFATc1, RANKL, and nuclear factor kappa-light-chain-enhancer of activated B cells p65 (NF-κB p65) (Santa Cruz Biotechnology, Dallas, TX, USA); Integrin-β3, phosphorylated extracellular signal-regulated kinase 1/2 (p-ERK1/2), phosphorylated c-Jun N-terminal kinase 1/2 (p-JNK1/2), phosphorylated p38 mitogen-activated protein kinase (p-p38), phosphorylated IκBα (p-IκBα), ERK1/2, JNK1/2, p38, and IκBα (Cell Signaling Technology, Danvers, MA, USA); and horseradish peroxidase-conjugated IgG (ENZO, Farmingdale, NY, USA). Visfatin was purchased from AdipoGen (San Diego, CA, USA). Macrophage colony-stimulating factor (M-CSF) and RANKL were purchased from PeproTech (Rocky Hill, CT, USA). OPG was purchased from R&D Systems (Minneapolis, MN, USA).

### 2.2. Visfatin Expression Analysis in Gene Expression Omnibus (GEO) Datasets

GEO datasets (GSE173078 and GSE230665 for periodontal diseases and OP, respectively) were obtained from the GEO (https://www.ncbi.nlm.nih.gov/geo (accessed on 1 August 2024)) to investigate visfatin expression in conditions associated with increased osteoclast activity. The GSE173078 dataset, which used gingival tissue samples, included non-smoking participants without diabetes, autoimmune diseases, or the need for antibiotic prophylaxis before dental procedures. The participants were divided into two main categories: 12 periodontally healthy individuals and 24 with periodontal disease (periodontitis and gingivitis). It provided expression values in fragments per kilobase of transcript per million (FPKM), which normalizes RNA-seq data for sequencing depth and gene length. The GSE230665 dataset included 15 participants: 3 healthy women as normal controls (NC) and 12 patients with primary OP. Femur tissue was collected from these patients for RNA extraction and microarray analysis. All participants met the “Recommended Diagnostic Criteria for OP in Chinese patients” and provided signed informed consent voluntarily. This dataset used log2 normalized expression to stabilize variance and normalize data distribution.

### 2.3. Mouse Bone Marrow-Derived Macrophage Preparation

All animal studies were conducted in accordance with the Guide for the Care and Use of Laboratory Animals (NIH publication No. 85-23, revised 1996) and were approved by the Institutional Animal Care and Use Committee at Pusan National University, Korea. Six-week-old C57BL/6 mice (Koatech, Pyeongtaek, South Korea) were used in this study. BMDMs were isolated from the whole bone marrow of mice, as described previously [38]. The harvested bone marrow cells were cultured in alpha minimum essential medium (α-MEM) (Life Technologies, Carlsbad, CA, USA) supplemented with 10% fetal bovine serum (FBS) (Gibco BRL, Gaithersburg, MD, USA), 1% penicillin/streptomycin (Gibco BRL), and M-CSF (20 ng/mL). After 3 d of incubation, the medium was replaced under the same conditions. Subsequently, the adherent cells, which had been cultured for a total of 7 days, were used as BMDMs for further analysis.

### 2.4. Osteoclast Differentiation and Tartrate-Resistant Acid Phosphatase Staining

Mouse BMDMs were cultured in 24-well plates at 37 °C and 5% CO_2_ in α-MEM supplemented with 10% FBS, 20 ng/mL M-CSF, and 100 ng/mL RANKL for 6 d, with the medium being replaced every 3 d. Control cells were treated with M-CSF only, without RANKL supplementation. Subsequently, the cells were stained for tartrate-resistant acid phosphatase (TRAP) using the TRACP & ALP double-staining kit (Takara, Shiga, Japan). TRAP-positive osteoclasts with at least three nuclei were counted in fields of 550 µm (width) by 375 µm (height). Four fields were used to generate each graphed dataset. The area of TRAP (+) cells was measured using ImageJ 1.15 software [39].

### 2.5. Resorption Pit Formation Assay

Resorption pit formation assay was performed on a bone resorption assay plate 24 (Cosmo bio, Tokyo, Japan). BMDMs were seeded onto 24-well plates at a density of 1 × 10^5^ and allowed to attach to the plates for 24 h at 37 °C, then cultured in α-MEM supplemented with stimulus for 6 days. The percentage of the resorbed areas of resorption pits were measured under microscopic examination using ImageJ. The assays were performed in triplicate, and a representative view from each assay is presented.

### 2.6. Western Blot Analysis

Harvested cells were lysed in radioimmunoprecipitation assay buffer (iNtRON Biotechnology, Sungnam, Republic of Korea) containing a protease inhibitor cocktail (Roche, Mannheim, Germany). Protein extracts (30 µg/lane) were separated using sodium dodecyl sulfate-polyacrylamide gel electrophoresis and transferred onto nitrocellulose membranes (Amersham Pharmacia Biotech, Little Chalfont, UK). Subsequently, the membranes were blocked with 5% skim milk in phosphate-buffered saline (PBS) containing 0.1% Tween 20 for 1 h at room temperature and probed with the appropriate antibodies. Protein blots on the membranes were visualized using an enhanced chemiluminescence detection system (Amersham Pharmacia Biotech, Little Chalfont, UK).

### 2.7. Enzyme-Linked Immunosorbent Assay

Mouse BMDMs were cultured in 24-well plates at 37 °C and 5% CO_2_ in α-MEM supplemented with 10% FBS, 30 ng/mL M-CSF, and 100 ng/mL RANKL for 1 and 3 d. The amount of secreted visfatin in the culture media was determined using an enzyme-linked immunosorbent assay, according to the manufacturer’s instructions (ENZO). The absorbance of the samples was monitored at 450 nm using a Victor X3, P multimode plate reader (Perkin Elmer, Hopkinton, MA, USA), and visfatin concentrations were determined by interpolating the values against a standard curve generated according to the manufacturer’s instructions.

### 2.8. Immunocytochemistry

Cells cultured on coverslips were fixed in 4% paraformaldehyde/PBS, blocked with 1% bovine serum albumin/PBS, and labeled with the appropriate primary antibodies, followed by incubation with Alexa Fluor 488-conjugated or Alexa Fluor 594-conjugated secondary antibodies. Subsequently, the coverslips were mounted on slides using a fluorescent mounting medium containing 4′,6-diamidino-2-phenylindole (Vector Labs, Burlingame, CA, USA). Next, the cells were analyzed using an LSM900 confocal microscope (ZEISS, Oberkochen, Germany).

### 2.9. Reverse-Transcription Quantitative Real-Time Polymerase Chain Reaction

Total RNA was isolated from BMDMs using TRIzol reagent (Invitrogen, Carlsbad, CA, USA). cDNA was synthesized from 1 μg of total RNA using a reverse transcription kit (iNtRON Biotechnology, Sungnam, South Korea). Quantitative PCR (qPCR) was performed using the SYBR^®^ Green method (Roche Applied Science, Penzberg, Upper Bavaria, Germany). The entire cycling process, including data analysis, was completed in less than 60 min and was monitored using Light Cycler software (version 4.0). The sequences of the oligonucleotide primers used for qPCR are shown Table 1.

### 2.10. Statistical Analysis

Data are presented as the mean ± standard deviation from at least three independent experiments. To compare two independent groups, a two-sample Student’s *t*-test was used. For comparisons among multiple groups, one-way analysis of variance (ANOVA) followed by Tukey’s honest significant difference (HSD) post hoc test was applied. A *p*-value of less than 0.05 was considered statistically significant, with significance levels indicated as * *p* < 0.05, ** *p* < 0.01, and *** *p* < 0.001.

## 3. Results

### 3.1. Visfatin Expression in Conditions with Elevated Osteoclast Activity

**Figure 1:**
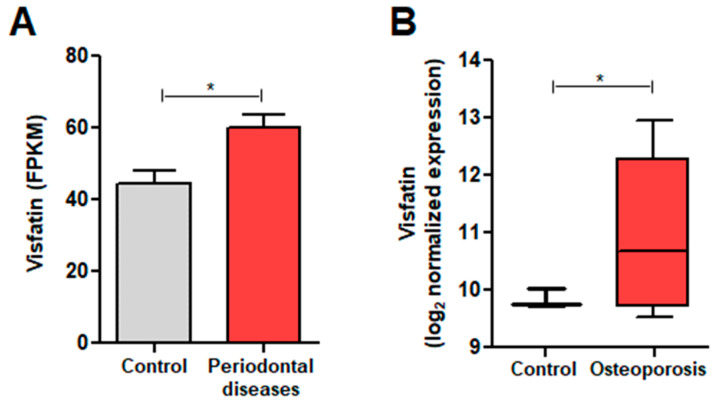
Visfatin expression analysis in periodontal diseases and osteoporosis (OP). (**A**) Visfatin expression in patients with periodontal disease compared to controls. (**B**) Visfatin expression in patients with OP compared to controls. The data are presented as box-and-whisker plots with the mean values indicated by an ‘x’. * *p* < 0.05.

GEO data were first analyzed to examine the relationship between visfatin expression and conditions with elevated osteoclast activity, such as periodontal diseases (gingivitis and periodontitis) and OP (Figure 1). In the periodontal disease dataset (GSE173078), visfatin expression was significantly higher in patients with periodontal disease (60.06 ± 18.61) than in controls (44.30 ± 13.60) (Figure 1A). Similarly, in the OP dataset (GSE230665), visfatin expression was higher in patients with OP (10.96 ± 1.32) than in controls (9.83 ± 0.16) (Figure 1B). These results suggest that visfatin is upregulated in conditions with elevated osteoclast activity.

### 3.2. Visfatin Increased NFATc1 Protein Levels

**Figure 2:**
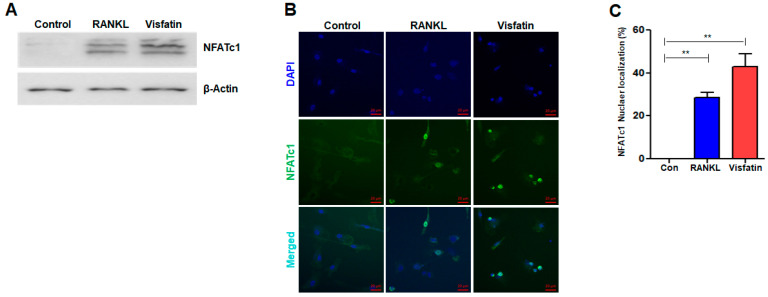
Effects of visfatin on NFATc1 expression and activation in BMDMs. BMDMs were treated with 100 ng/mL RANKL or 500 ng/mL visfatin for 24 h. (**A**) Western blotting quantified the protein levels of NFATc1. (**B**) Immunocytochemistry visualized NFATc1 nuclear localization with fluorescence microscopy. Nuclei were counterstained with 4’,6-diamidino-2-phenylindole (DAPI: blue). (**C**) Quantitative results for the percentage of nuclear localization of NFATc1. ** *p* < 0.01.

We hypothesized that visfatin upregulation in periodontal diseases and OP was relevant for inducing osteoclast differentiation. To test this hypothesis, the regulation of NFATc1 expression was studied in our culture system. NFATc1 is essential for RANKL-induced osteoclast differentiation, as it activates key genes involved in this process [9]. RANKL treatment upregulated NFATc1 protein levels, thereby validating the activation of the RANKL-NFATc1 signaling pathway in the BMDMs (Figure 2A). Next, the effect of visfatin on NFATc1 expression in BMDMs was evaluated. NFATc1 protein levels in visfatin-treated BMDMs were significantly elevated compared with those in control cells, mirroring the effects observed with RANKL (Figure 2A). Furthermore, immunocytochemical analysis revealed an increase in the proportion of NFATc1-positive cells following visfatin treatment, comparable to the increase observed in RANKL-treated cells (Figure 2B,C). These results raise the question of whether the upregulation of NFATc1 levels is associated with osteoclast differentiation.

### 3.3. Visfatin-Induced Osteoclast Differentiation Was RANKL-Dependent

**Figure 3:**
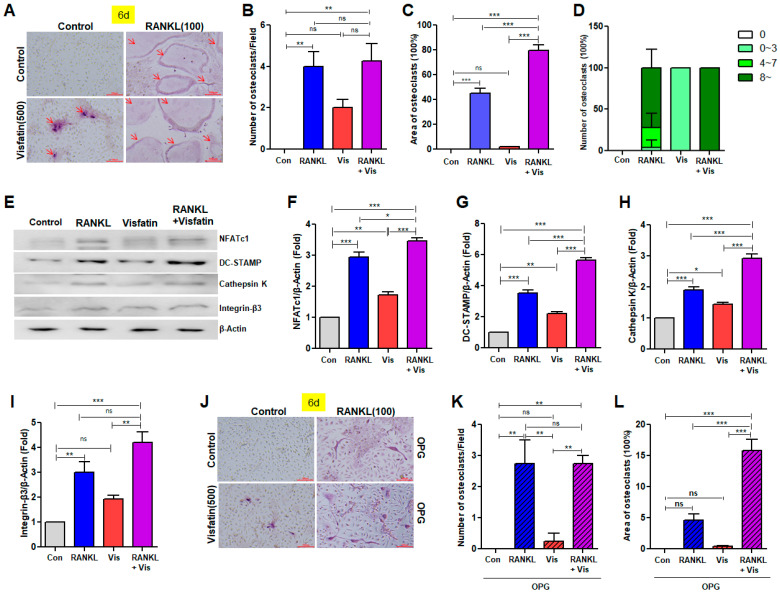
Effects of visfatin on osteoclast differentiation in BMDMs. (**A**–**I**) BMDMs were treated with 100 ng/mL RANKL and/or 500 ng/mL visfatin for 6 d. (**A**) TRAP staining was performed to determine differentiated osteoclasts (arrow; original magnification, ×200). (**B**) Counted number of TRAP-positive multinucleated cells (MNCs: ≥3 nuclei). (**C**) Area of TRAP-positive MNCs (≥3 nuclei) measured using ImageJ software. (**D**) Calculation of percentage of cells with the specified range of nuclei per cells. (**E**) Western blotting quantified protein levels of NFATc1, DC-STAMP, Cathepsin K, and Integrin-β levels, with β-Actin as a control. (**F**–**I**) Densitometric analysis of NFATc1 (**F**), DC-STAMP (**G**), Cathepsin K (**H**), and Integrin-β3 (**I**) normalized to β-Actin. (**J**–**L**) BMDMs were treated with 100 ng/mL RANKL and/or 500 ng/mL visfatin in presence of 100 ng/mL OPG for 6 d. (**J**) TRAP staining identified differentiated osteoclasts (×200). (**K**,**L**) Quantification of number and area of TRAP-positive MNCs (≥3 nuclei). ns: not significant. * *p* < 0.05, ** *p* < 0.01, *** *p* < 0.001.

To induce osteoclast differentiation, BMDMs were cultured with RANKL and/or visfatin in combination with M-CSF for 6 d. Subsequently, TRAP staining was performed to evaluate the presence of multinucleated osteoclasts. RANKL, a positive control stimulator of osteoclastogenesis, effectively induced the differentiation of TRAP(+) multinucleated osteoclasts (Figure 3A–D and Figure S1). Visfatin also increased the number of TRAP(+) cells relative to that of the control, albeit to a lesser extent than RANKL (Figure 3A,C and Figure S1). Notably, visfatin treatment significantly increased both the number and size of RANKL-induced TRAP(+) multinucleated osteoclasts (Figure 3A–D and Figure S1). Although no significant difference was initially observed between the RANKL group and the RANKL + visfatin cotreatment group (Figure 3B), analysis based on the number of nuclei revealed that the percentage of multinucleated osteoclast cells possessing more than eight nuclei was increased in the cotreated group (Figure 3D). Next, we examined the effects of RANKL, with or without visfatin, on the expression of several osteoclast marker genes. RANKL increased the protein levels of NFATc1 and DC-STAMP, a gene involved in osteoclast fusion, and visfatin treatment significantly enhanced the levels of RANKL-induced DC-STAMP proteins (Figure 3E–G). RANKL also upregulated the expression level of Cathepsin K and Integrin-β3 proteins, with an increase observed upon visfatin addition (Figure 3E,H,I)). Visfatin treatment alone resulted in elevated expression levels of NFATc1, DC-STAMP, Cathepsin K, and Integrin-β3 proteins compared with the levels observed in the control group; however, the extent of this increase was less pronounced than that observed with RANKL treatment (Figure 3E–I). OPG, a decoy receptor for RANKL, inhibits RANKL-mediated osteoclastogenesis by disrupting the binding of RANKL to RANK [7,11]. Therefore, to investigate whether visfatin-induced osteoclast differentiation is associated with RANKL/RANK signaling, cells were treated with OPG. OPG effectively inhibited RANKL- and visfatin-induced osteoclastogenesis, as well as osteoclast differentiation induced by co-treatment with RANKL and visfatin (Figure 3J–L compared to Figure 3A–C).

### 3.4. Visfatin Enhanced RANKL-Induced Osteoclast Differentiation

**Figure 4:**
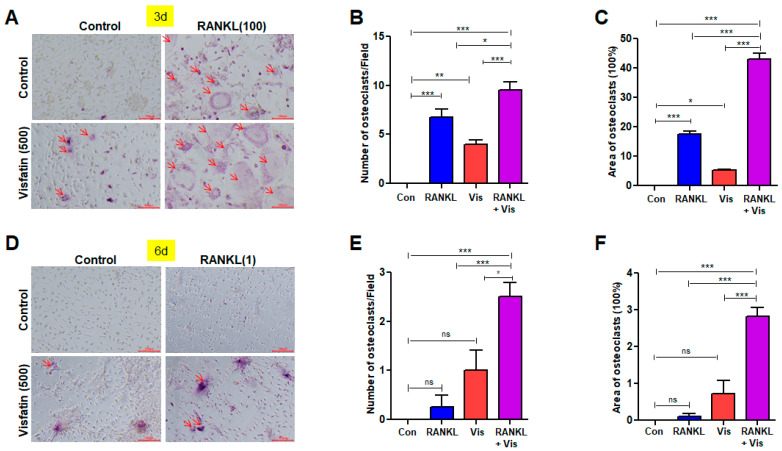
Effects of visfatin on RANKL-induced osteoclast differentiation in BMDMs. (**A**–**C**) BMDMs were treated with 100 ng/mL RANKL and/or 500 ng/mL visfatin for 3 d. (**A**) TRAP staining identified osteoclasts (arrow; ×200). (**B**,**C**) Quantification of number and area of TRAP-positive MNCs (≥3 nuclei). **(D**–**F**) BMDMs were treated with 1 ng/mL RANKL and/or 500 ng/mL visfatin for 6 d. (**D**) TRAP staining identified differentiated osteoclasts (arrow: ×200). (**E**,**F**) Quantification of number and area of TRAP-positive MNCs (≥3 nuclei). ns: not significant. * *p* < 0.05, ** *p* < 0.01, *** *p* < 0.001.

To investigate the effect of visfatin in enhancing RANKL-induced osteoclast differentiation, TRAP staining was performed after 3 d of osteoclastogenic induction (Figure 4A and Figure S2A). Treatment with 100 ng/mL RANKL resulted in a significantly higher formation of TRAP(+) multinucleated osteoclasts than the control treatment (Figure 4A–C and Figure S2A). However, the area and size of TRAP(+) multinucleated osteoclasts were notably reduced at the 3 d mark compared with their counterparts after 6 d of culture (Figure 3A,C vs. Figure 4A,C). After treatment with visfatin alone for 3 d, only a few TRAP(+) cells were observed (Figure 4A–C and Figure S2A). In contrast, co-treatment with RANKL and visfatin for 3 d led to an increase in both the number and size of TRAP(+) multinucleated osteoclasts, surpassing the effects of either visfatin or RANKL alone (Figure 4A–C) and reaching levels comparable to those observed after 6 d of treatment with RANKL alone (Figure 3A,C vs. Figure 4A,C). Next, we investigated the effect of visfatin on RANKL at a concentration of 1 ng/mL, which is insufficient for inducing osteoclast differentiation. TRAP staining was performed after 6 d of osteoclastogenic induction (Figure 4D and Figure S2B). Although RANKL at 1 ng/mL alone did not induce osteoclast formation in BMDMs, co-treatment with visfatin resulted in a higher formation of TRAP(+) multinucleated osteoclasts compared with that observed in the other groups (Figure 4D–F and Figure S2B).

### 3.5. Visfatin Blockade Attenuated RANKL-Induced Osteoclast Differentiation

**Figure 5:**
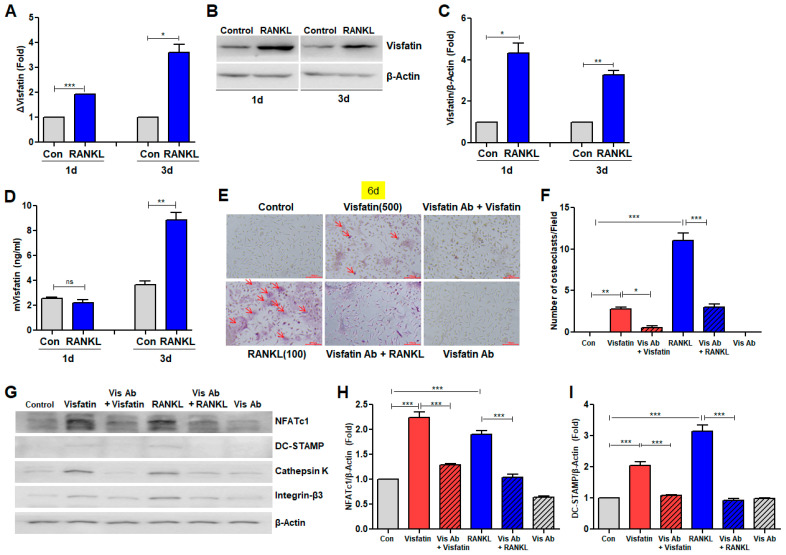

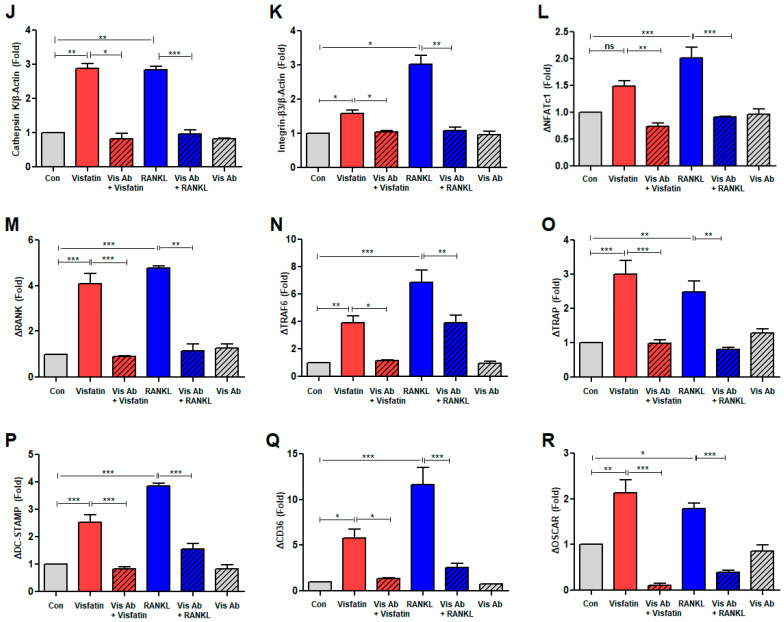
Effects of visfatin blockade on RANKL-induced osteoclast differentiation in BMDMs. (**A**–**D**) BMDMs were treated with 100 ng/mL RANKL for 1 and 3 d. (**A**) Real-time reverse transcription PCR (RT-PCR) was performed to quantify mRNA levels of visfatin. Expression level of the control (untreated) was set to 1, and values were normalized to β-Actin mRNA levels. (**B**) Western blotting quantified protein levels of visfatin, with β-Actin as a control. (**C**) Densitometric analysis of visfatin protein levels, normalized to β-Actin. (**D**) Secreted levels of visfatin protein in medium were measured using enzyme-linked immunosorbent assay. (**E**–**R**) BMDMs were treated with 100 ng/mL RANKL or 500 ng/mL visfatin for 6 d, in presence or absence of anti-visfatin neutralizing antibodies (2 μg/mL). (**E**) TRAP staining identified osteoclasts (arrow; ×200). (**F**) Quantification of number of TRAP-positive MNCs (≥3 nuclei). (**G**) Western blotting quantified protein levels of NFATc1, DC-STAMP, Cathepsin K, and Integrin-β, with β-Actin as a control. (**H**–**K**) Densitometric analysis of NFATc1 (H), DC-STAMP (**I**), Cathepsin K (**J**), and Integrin-β3 (**K**) normalized to β-Actin. (**L**–**R**) Real-time RT-PCR quantified mRNA levels of osteoclast markers, normalized to β-Actin. ns: not significant. * *p* < 0.05, ** *p* < 0.01, *** *p* < 0.001.

The expression pattern of the visfatin gene during RANKL-induced osteoclast differentiation was investigated. BMDMs were cultured with RANKL for 1 and 3 d. Both the mRNA and protein levels of visfatin were significantly increased by the treatment of RANKL compared with those in the control group (Figure 5A–D). Given that RANKL upregulated visfatin expression, the role of visfatin in RANKL-induced osteoclast differentiation was explored further. BMDMs were co-treated with neutralizing anti-visfatin antibodies alongside RANKL. This co-treatment effectively reversed the RANKL-induced increase in the number of multinucleated TRAP(+) cells (Figure 5E,F and Figure S3). The specificity of the neutralizing antibodies was confirmed by their ability to inhibit visfatin-induced osteoclast differentiation (Figure 5E,F and Figure S3). The effects of these treatments on the protein expression of osteoclast markers were also assessed. RANKL treatment resulted in a significant upregulation of NFATc1, DC-STAMP, Cathepsin K, and Integrin-β3 protein levels (Figure 5G–K). However, co-treatment with neutralizing anti-visfatin antibodies and RANKL significantly attenuated this upregulation, reducing the protein levels to levels comparable to those in the control group (Figure 5G–K). Treatment with visfatin alone also increased the protein levels of NFATc1, DC-STAMP, Cathepsin K, and Integrin-β3 compared with those in the control group (Figure 5G–K). However, the visfatin-induced elevation of the expression of these osteoclast marker proteins was effectively reversed by the neutralizing anti-visfatin antibodies (Figure 5G–K). Furthermore, qPCR analysis revealed that RANKL treatment significantly increased the mRNA levels of several osteoclast marker genes, including NFATc1, RANK, TRAF6, TRAP, DC-STAMP, CD-36, and OSCAR (Figure 5L–R). However, this increase was mitigated by treatment with neutralizing anti-visfatin antibodies (Figure 5L–R). Similarly, visfatin treatment induced the expression of these osteoclast marker genes; however, the administration of anti-visfatin neutralizing antibodies significantly reduced the visfatin-induced increase in their mRNA levels (Figure 5L–R).

### 3.6. Visfatin Blockade Inhibited RANKL-Induced Activation of Mitogen-Activated Protein Kinase and NF-kB Signaling Pathways

**Figure 6:**
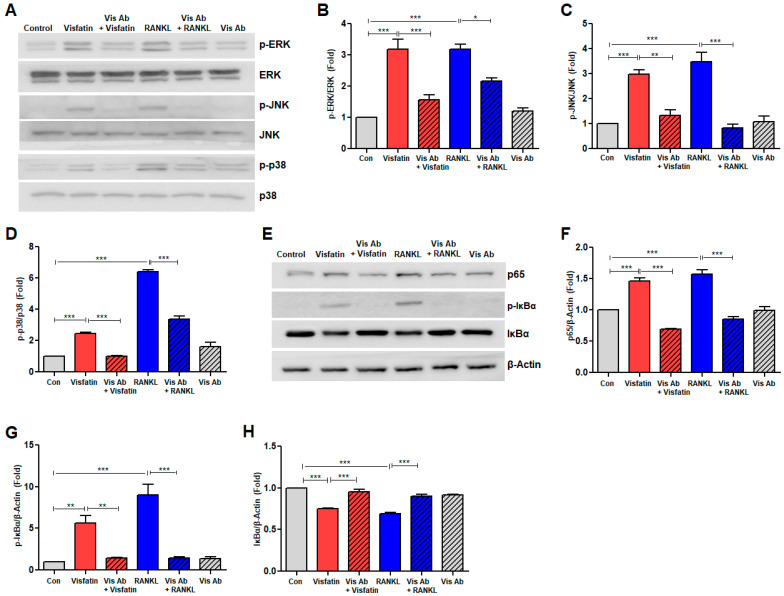
Effects of visfatin blockade on RANKL-induced activation of NF-kB and MAPK signaling pathways in BMDMs. BMDMs were treated with 100 ng/mL RANKL or 500 ng/mL visfatin for 20 min, in presence or absence of anti-visfatin neutralizing antibodies (2 μg/mL). (**A**) Western blotting quantified protein levels of phospho-ERK1/2, ERK1/2, phospho-JNK1/2, JNK1/2, phospho-p38 MAPK, and p38 MAPK, with β-Actin as a control. (**B**–**D**) Densitometric analysis for assessment of relative levels of phosphorylated proteins to total MAPK protein levels. (**E**) Western blotting quantified protein levels of NF-κB p65, phospho-IκBα, and IκBα, with β-Actin as a control. (**F**–**H**) Densitometric analysis of p65 (**F**), p-IκBα (**G**), and IκBα (**H**), normalized to β-Actin. * *p* < 0.05, ** *p* < 0.01, *** *p* < 0.001.

Given the critical roles of MAPKs, such as ERK, JNK, and p38 MAPK, alongside the NF-κB signaling pathway in regulating RANKL signaling [7,9], we investigated the role of visfatin blockade in modulating these pathways during RANKL-induced osteoclastogenesis in BMDMs. RANKL stimulation significantly increased the phosphorylation of ERK1/2, JNK1/2, and p38 MAPK; however, the total levels of ERK, JNK, and p38 remained unchanged (Figure 6A–D). Notably, this phosphorylation was effectively inhibited by pretreatment with neutralizing anti-visfatin antibodies (Figure 6A–D). Additionally, RANKL treatment increased the levels of NF-κB p65 protein and induced the phosphorylation of serine 32 in IκBα (phospho-IκBα), accompanied by a reduction in total IκBα protein levels (Figure 6E–H). However, these RANKL-induced changes were reversed following treatment with neutralizing anti-visfatin antibodies (Figure 6E–H). Notably, similar phosphorylation and protein level alterations were observed following treatment with visfatin (Figure 6A–H).

### 3.7. Visfatin Enhances the Resorptive Activity of RANKL-Induced Osteoclasts, While Visfatin Blockade Attenuates This Activity

**Figure 7:**
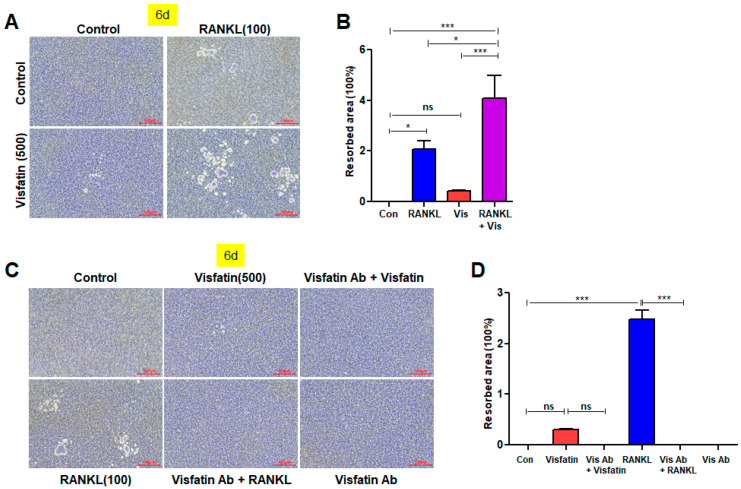
Effects of visfatin on the resorptive activity of RANKL-induced osteoclasts in BMDMs. (**A**,**B**) BMDMs were treated with 100 ng/mL RANKL and/or 500 ng/mL visfatin for 6 d. (**C**,**D**) BMDMs were treated with 100 ng/mL RANKL or 500 ng/mL visfatin for 20 min, in presence or absence of anti-visfatin neutralizing antibodies (2 μg/mL). (**A**,**C**) Bone resorption assay was performed to determine resorptive activity of osteoclasts (original magnification, ×200). (**B**,**D**) Resorbed areas of the resorption pits measured using ImageJ. ns: not significant. * *p* < 0.05, *** *p* < 0.001.

To investigate the effect of visfatin on resorptive activity of RANKL-induced osteoclasts, we performed a bone resorption assay. Both RANKL and visfatin treatments increased the degree of resorption pit formation, although the effect of visfatin was less pronounced compared to RANKL. However, when both treatments were applied together, a significant increase in the area of resorption pits was observed, suggesting that visfatin exerts a synergistic effect on RANKL-induced osteoclast activity (Figure 7A,B). Furthermore, treatment with an anti-visfatin neutralizing antibody reduced the extent of resorption pit formation induced by visfatin and also attenuated the effect of RANKL (Figure 7C,D). This reduction in osteoclast activity upon visfatin blockade underscores the role of visfatin not only in RANKL-induced osteoclast differentiation but also in the overall activation of osteoclastogenesis (Figure 7C,D).

## 4. Discussion

Our findings reveal that visfatin-induced osteoclast differentiation is associated with increased NFATc1 expression. This upregulation of NFATc1 in response to visfatin aligns with recent evidence suggesting that visfatin can epigenetically regulate NFATc1 transcription in the murine macrophage RAW 264.7 cell line [36]. Given that RANKL-induced osteoclastogenesis is considered a key canonical pathway, often described as a RANKL-dependent mechanism [9], its regulation is significant. Notably, this process can be inhibited by OPG, a soluble decoy receptor for RANKL, by competitively binding to RANKL, thereby preventing RANKL from binding to its receptor, RANK, and blocking RANKL-induced osteoclastogenesis [11]. In the present study, visfatin-induced osteoclast differentiation was significantly reduced in the presence of OPG, indicating that this differentiation process is dependent on the RANKL signaling pathway. Therefore, it is plausible that visfatin acts as an osteoclastogenic adipokine, at least in part, through the RANKL-NFATc1 signaling axis.

Elucidating the mechanisms by which visfatin modulates bone metabolism is essential for understanding its role in skeletal health and developing targeted therapies aimed at mitigating bone loss-related disorders characterized by dysregulated osteoclast activity, such as OP and periodontitis-associated bone resorption [1,3,4]. Visfatin exerts its biological effects through intricate signaling pathways, including the NF-κB and MAPK pathways, which play critical roles in mediating inflammatory responses and various cellular processes [22,23,24]. Our previous studies have demonstrated that visfatin activates NF-κB and MAPKs, influencing inflammation, cancer cell proliferation, vascular angiogenesis, and cellular senescence [31,32,40,41,42]. In the present study, visfatin-induced osteoclast differentiation was associated with the activation of NF-κB and MAPKs, representing a hallmark gene activation profile driving osteoclastogenesis. Osteoclast differentiation is triggered by RANKL binding to RANK, leading to the activation of multiple signaling pathways, including NF-κB and MAPK pathways, which subsequently induce NFATc1 activation and the expression of osteoclast-specific genes [7,8,9]. Therefore, our findings suggest that visfatin may promote osteoclast differentiation through the RANKL-NF-κB/MAPKs-NFATc1 signaling axis.

Furthermore, although visfatin alone induced relatively modest osteoclast differentiation compared to RANKL, it significantly enhanced osteoclast differentiation, even at sub-osteoclastogenic concentrations of RANKL, as well as the optimal osteoclastogenic concentration. These findings suggest a synergistic relationship between visfatin and RANKL in promoting osteoclastogenesis. Similar synergistic effects with RANKL have been reported for tumor necrosis factor-alpha, interleukin 1 (IL)-1, and IL-15 [43,44,45]. However, a key distinction is that, unlike visfatin, these cytokines induce osteoclast formation only in the presence of permissively low levels of RANKL. Notably, the combination of IL-6 and its soluble receptor (sIL-6R) leads to a weak yet significant increase in osteoclast formation, enhancing RANKL-induced osteoclast differentiation at low RANKL concentrations while inhibiting it at high concentrations [46]. This differential regulation of osteoclastogenesis by IL-6/sIL-6R can be attributed to varying degrees of activation of the NF-κB and MAPK pathways in response to different RANKL concentrations. In contrast, visfatin consistently promotes osteoclast differentiation. Specifically, the extent of osteoclast differentiation in the presence of visfatin and RANKL increases with both higher RANKL concentrations and longer culture durations, although visfatin promotes osteoclast differentiation even at low RANKL levels. Our findings indicate that high visfatin expression correlates with periodontal diseases and OP and that RANKL upregulated visfatin gene expression in BMDMs, suggesting that increased visfatin levels could serve as a key trigger of osteoclast differentiation, even under conditions of low RANKL concentrations. This mechanism may contribute to the pathogenesis of destructive inflammatory bone diseases, including inflammatory rheumatic diseases, osteoarthritis, cancer, aging, obesity, periodontitis, and OP. Supporting this hypothesis, a growing body of evidence has implicated visfatin in the etiology of these bone loss-associated conditions [28,31,32,33,34,35,36,47,48,49].

Visfatin (extracellular NAMPT) plays a crucial role in various biological processes, including osteoclast differentiation, but the mechanisms underlying its action remain unclear. A major challenge is the lack of a specific receptor for extracellular visfatin, which complicates our understanding of its signaling pathways. While potential receptors, such as IR and TLR4 [25,26,27,32], have been suggested, the exact mechanism of visfatin’s action is still not fully understood. In our preliminary studies, we focused on the role of TLR4 signaling in osteoclast differentiation. Using the TLR4 inhibitor TAK242 [50], we observed a significant reduction in osteoclast differentiation following RANKL stimulation. This suggests that TLR4 is involved in osteoclastogenesis, and we hypothesize that visfatin, produced in response to RANKL, interacts with TLR4 to promote osteoclast differentiation. Further experiments are needed to confirm this hypothesis and clarify the underlying molecular mechanisms. Another key aspect of visfatin biology is its potential enzymatic activity. Visfatin shares the same amino acid sequence with intracellular NAMPT (iNAMPT), an essential enzyme in NAD+ biosynthesis. However, it remains unclear whether extracellular visfatin retains enzymatic activity. To investigate the relationship between visfatin’s enzymatic function and its effects on osteoclast differentiation, we used FK866, a known NAMPT inhibitor [51]. Unfortunately, FK866 induced cell death in BMDMs, which complicated the interpretation of visfatin’s role in osteoclast differentiation. Despite this, visfatin may regulate osteoclast differentiation through TLR4 receptor signaling and/or its enzymatic activity in NAD+ biosynthesis. Further research is needed to clarify which mechanism, or both, mediate these effects. Understanding visfatin’s role in osteoclastogenesis will provide valuable insights into its broader functions in bone metabolism and related diseases.

The discrepancy between our results and those of Baek et al. regarding the effects of visfatin on osteoclast differentiation may be attributed to differences in mouse strains and variations in experimental conditions. While we observed that visfatin promoted osteoclast differentiation in C57BL/6 mice-derived BMDMs, Baek et al. reported inhibition of differentiation in ICR mice-derived BMDMs [37]. Previous studies have highlighted strain-specific differences in cellular responses: C57BL/6J and ICR mice exhibit strain-dependent reactivity to cigarette smoke, with differential regulation of HDAC2 and NF-κB pathways [52]; BMMs from ICR mice express S1PR1, S1PR2, and S1PR3, while C57BL/6 BMMs do not express S1PR3, suggesting strain-specific variations in BMDMs [53]; and strain-dependent differences in NIL-3 expression were observed, indicating that the regulatory mechanisms of hematopoiesis differ between C57BL/6 and ICR mice [54]. These factors, along with differences in BMDM culture conditions and administration protocols, may contribute to the observed discrepancies in osteoclast differentiation outcomes. Further research is needed to clarify the strain-specific molecular pathways responsible for these contrasting effects.

OP is a prevalent skeletal disorder characterized by reduced bone density and increased fracture risk, largely driven by an imbalance between bone formation and resorption [15,55]. In OP, dysregulated RANKL signaling leads to increased osteoclast activity and subsequent bone loss [10,56]. In addition to bisphosphonates, denosumab, a well-established therapeutic antibody that neutralizes RANKL, has demonstrated significant efficacy in clinical trials, particularly in reducing the risk of vertebral fractures in postmenopausal women with OP [2,3,57]. Our study demonstrated that the neutralization of visfatin using blocking antibodies resulted in the inhibition of RANKL-induced osteoclastogenesis, suggesting that visfatin signaling may serve as a novel mediator linking RANKL to osteoclastogenesis. This underscores the potential of visfatin signaling inhibitors, including neutralizing antibodies, as a therapeutic strategy OP and other bone resorption-related diseases, such as periodontal disease. However, while in vitro studies have provided valuable insights into the cellular mechanisms underlying visfatin’s role in bone diseases, they do have limitations. Specifically, in vitro experiments allow for controlled examination of cellular responses to specific treatments, but they do not capture the complexity of in vivo environments, where systemic, hormonal, and cellular factors influence osteoclast differentiation and activity. These factors may lead to different outcomes due to interactions that are absent in vitro. Therefore, further in vivo studies are needed to determine whether the conditions of our in vitro experiments, including the concentration of visfatin used, accurately reflect the physiological context in vivo. Additionally, in vivo studies are essential to assess the safety, efficacy, and biological interactions of visfatin-neutralizing antibodies in a more complex system, especially given the complexities of osteoporosis and the potential for unforeseen effects. 

## 5. Conclusions

This study identified visfatin as an inductive modulator of osteoclastogenesis. Visfatin acts synergistically with RANKL to induce osteoclast differentiation and activation. In addition, our findings underscore the distinctive role of visfatin in the direct mediation of RANKL-induced osteoclastogenesis, extending beyond its established inflammatory functions. These findings suggest that dysregulation of visfatin signaling may represent a risk factor for bone loss-related diseases, including periodontal disease and OP.

## Figures and Tables

**Table 1:** The oligonucleotide primer sequences used for PCR.

Gene	Accession Number	Primer Sequence
Visfatin (756 bp)	NM_021524.2	Forward: 5′- GGCCACAAATTCCAGAGAACAG-3′
		Reverse: 5′- CCAAATGAGCAGATGCCCCTA -3′
NFATc1 (188 bp)	NM_001164110.1	Forward: 5′- CCAGTATACCAGCTCTGCCA-3′
		Reverse: 5′- GTGGGAAGTCAGAAGTGGGT-3′
RANK (181 bp)	NM_009399.5	Forward: 5′- AGAAGACGGTGCTGGAGTCT-3′
		Reverse: 5′- TAGGAGCAGTGAACCAGTCG-3′
TRAF6 (151 bp)	NM_009424.3	Forward: 5′- GCCCAGGCTGTTCATAATGT-3′
		Reverse: 5′- TCGCCCACGTACATACTCTG-3′
TRAP (190 bp)	NM_001102405.1	Forward: 5′- CAGTTGGCAGCAGCCAAGGAG-3′
		Reverse: 5′- TCCGTGCTCGGCGATGGACC-3′
DC-STAMP (264 bp)	NM_029422.4	Forward: 5′- CTAGCTGGCTGGACTTCATCC-3′
		Reverse: 5′- TCATGCTGTCTAGGAGACCTC-3′
CD36 (99 bp)	NM_001159558.2	Forward: 5′- TCCTCTGACATTTGCAGGTCTA-3′
		Reverse: 5′- AAAGGCATTGGCTGGAAGAA-3′
OSCAR (310 bp)	NM_175632	Forward: 5′- CTGCTGGATACGGATCAGCTCCCCAGA-3′
		Reverse: 5′- CCAAGGAGCCAGAACCTTCGAAACT-3′
GAPDH (139 bp)	NM_001289726.2	Forward: 5′- GGAGAAACCTGCCAAGTATG-3′
		Reverse: 5′- GAAGAGTGGGAGTTGCTGTT-3′

## Data Availability

The data presented in this study are available on request from the corresponding author.

## Supplementary Materials

The following supporting information can be downloaded at: https://www.mdpi.com/article/10.3390/biom14121500/s1.
