# Supplementary material for: Visfatin Enhances RANKL-Induced Osteoclastogenesis In Vitro: Synergistic Interactions and Its Role as a Mediator in Osteoclast Differentiation and Activation
Source: Biomolecules. 2024 Nov 25;14(12):1500. doi: 10.3390/biom14121500 (PMC11673010; doi:10.3390/biom14121500)
Supplement: Supplementary file 1 [file biomolecules-14-01500-s001.zip › Supplementary Figures-biomolecules-3291514.pdf]

Supplementary Figures

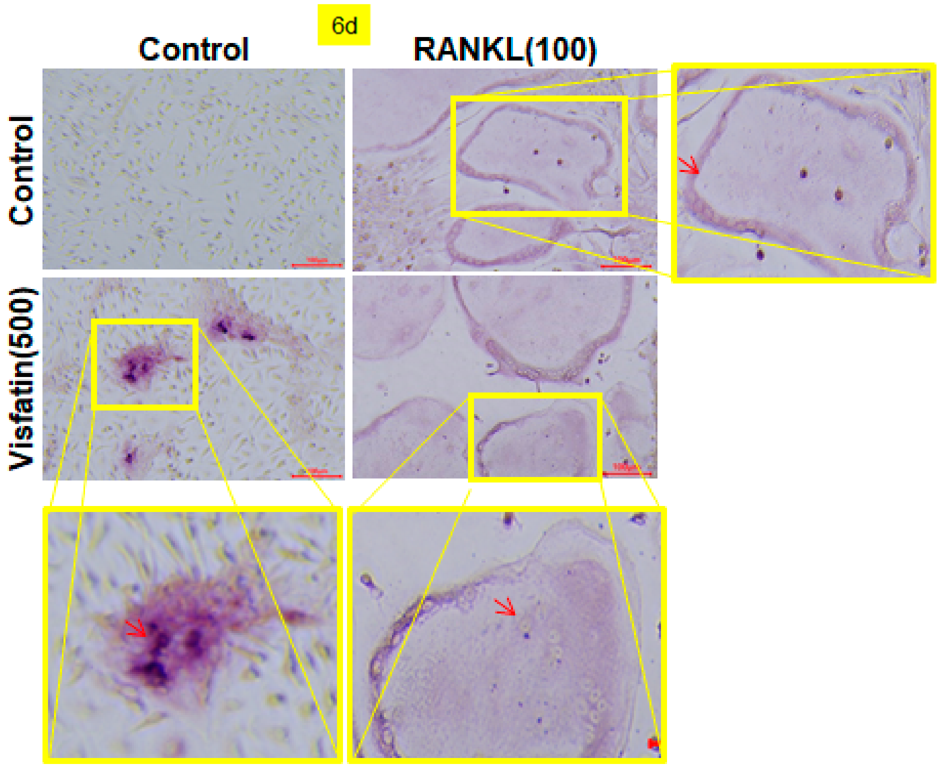

Figure S1: Enlarged view of the boxed area in Figure 3A**.** Arrow indicates TRAP-positive multinucleated osteoclast (original magnification, ×200)

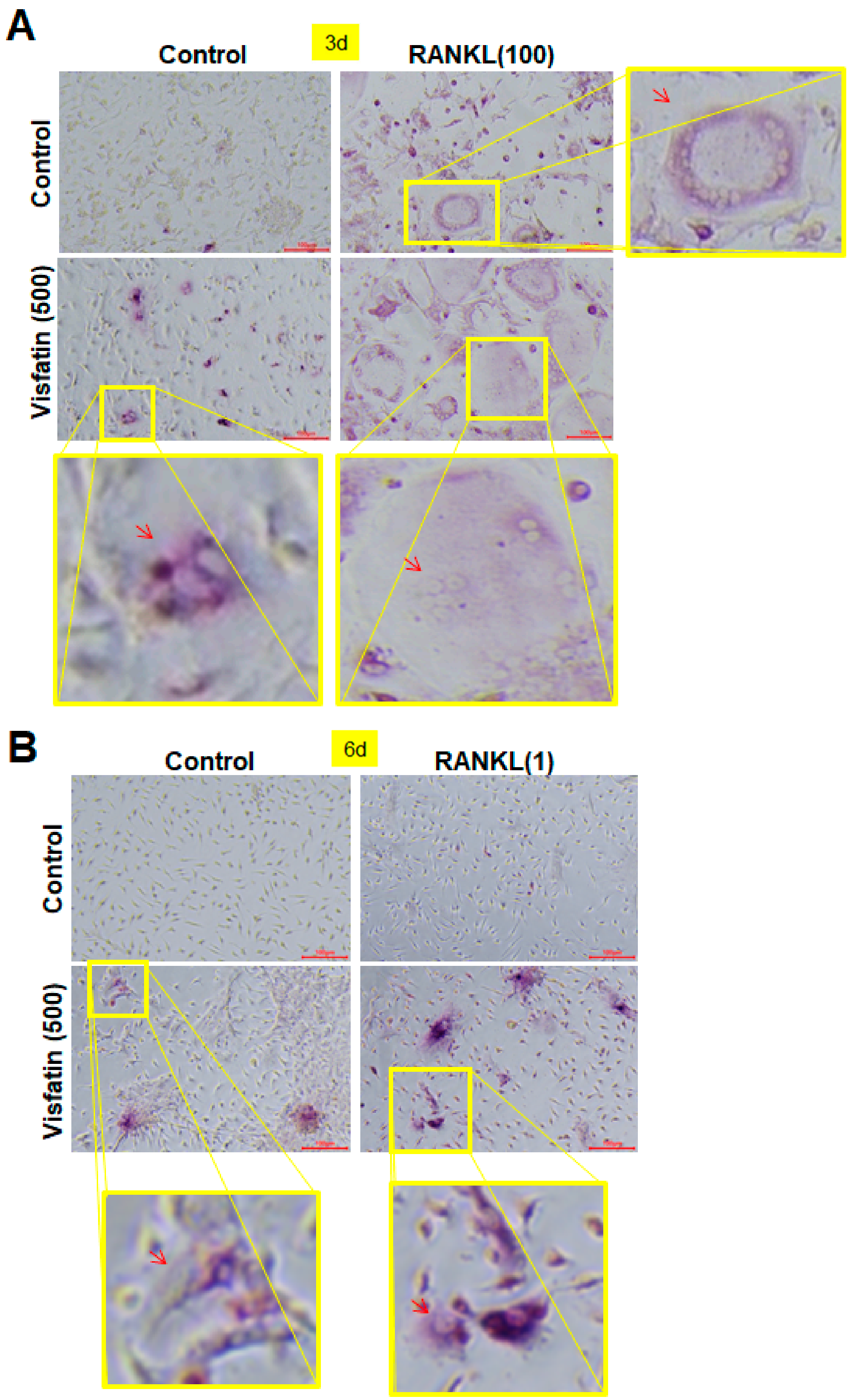

Figure S2: Enlarged view of the boxed area in Figure 4A (A) and 4D (B)**.** Arrow indicates TRAP-positive multinucleated osteoclast (original magnification, ×200)

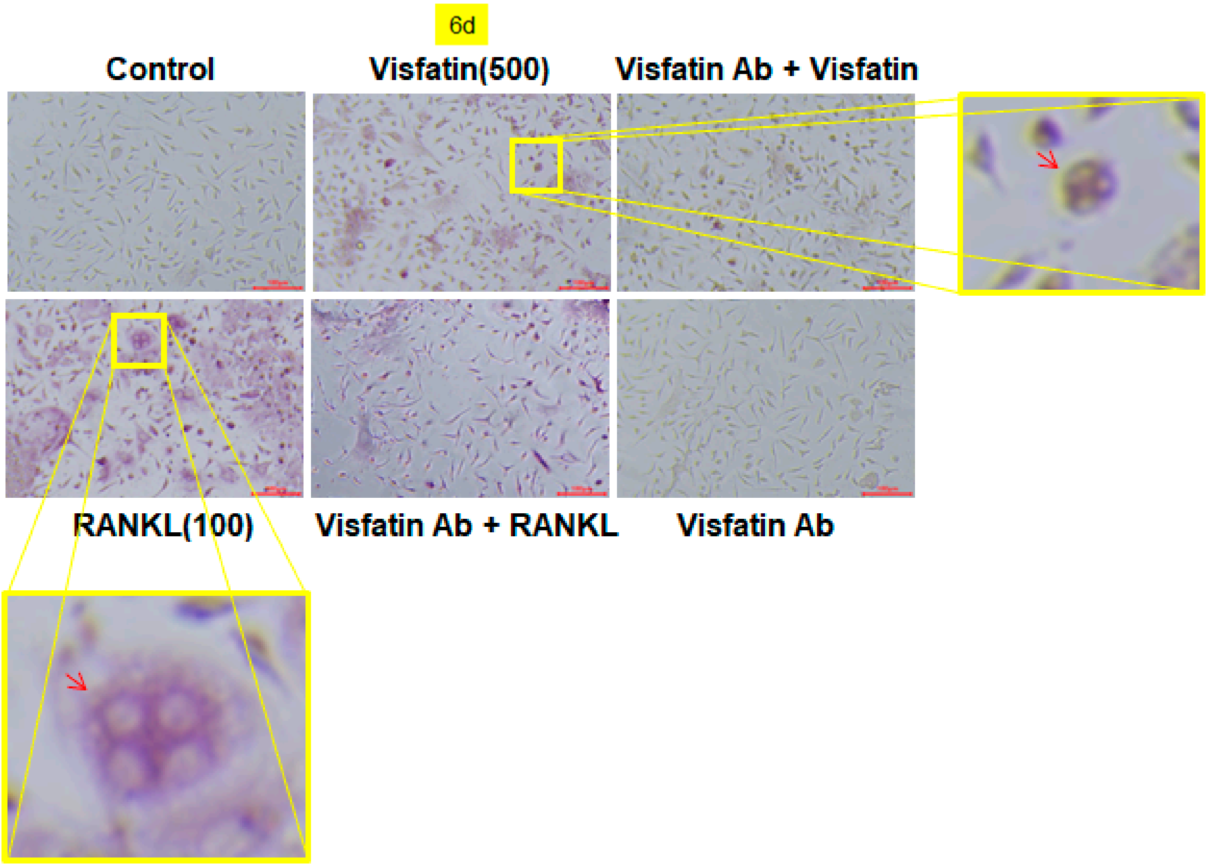

Figure S3: Enlarged view of the boxed area in Figure 5E**.** Arrow indicates TRAP-positive multinucleated osteoclast (original magnification, ×200)
